# Functional plasticity of neutrophils after low- or high-dose irradiation in cancer treatment – A mini review

**DOI:** 10.3389/fimmu.2023.1169670

**Published:** 2023-03-30

**Authors:** Jing Hu, Mingyue Pan, Yixi Wang, Yujie Zhu, Meidan Wang

**Affiliations:** ^1^ National Cancer Center/National Clinical Research Center for Cancer/Cancer Hospital & Shenzhen Hospital, Chinese Academy of Medical Sciences and Peking Union Medical College, Shenzhen, China; ^2^ Faculty of Law, University of Freiburg, Freiburg, Germany; ^3^ Department of Rehabilitation Medicine, Chongqing University Jiangjin Hospital, Chongqing University, Chongqing, China; ^4^ Department of Obstetrics and Gynecology, Nanjing University Medical School Affiliated Nanjing Drum Tower Hospital, Nanjing, Jiangsu, China; ^5^ Faculty of Biology, University of Freiburg, Freiburg, Germany

**Keywords:** neutrophils, low-dose radiotherapy, high-dose radiotherapy, tumor-associated neutrophils, cancer, tumor

## Abstract

Over the last several decades, radiotherapy has been considered the primary treatment option for a broad range of cancer types, aimed at prolonging patients’ survival and slowing down tumor regression. However, therapeutic outcomes of radiotherapy remain limited, and patients suffer from relapse shortly after radiation. Neutrophils can initiate an immune response to infection by releasing cytokines and chemokines to actively combat pathogens. In tumor immune microenvironment, tumor-derived signals reprogram neutrophils and induce their heterogeneity and functional versatility to promote or inhibit tumor growth. In this review, we present an overview of the typical phenotypes of neutrophils that emerge after exposure to low- and high-dose radiation. These phenotypes hold potential for developing synergistic therapeutic strategies to inhibit immunosuppressive activity and improve the antitumor effects of neutrophils to render radiation therapy as a more effective strategy for cancer patients, through tumor microenvironment modulation.

## 1 Introduction

Radiotherapy, a therapy that has been applied worldwide for several decades to reduce tumor size and relieve pain from metastatic cancer patients. Two-thirds of cancer patients benefit from radiation which delivers high-energy X-rays or particles to destroy tumors (1). There is ample evidence that high-dose radiotherapy is associated with prolonged overall survival, as demonstrated by a broad spectrum of cancer patients’ clinical therapeutic results and preclinical experiments. Patients who received low-dose irradiation following high-dose irradiation at local tumor triggered a systematic antitumor response rate of metastatic burden, as observed in preclinical studies (2, 3). So far, many studies demonstrated that high-dose irradiation triggers system toxicity and boosts antitumor immunity in cancer patients; low-dose irradiation, however, is under more investigation especially its influence on profoundly reprogramming the tumor immune microenvironment (TIME) and cytotoxic T cell recruitment which can effectively reverse cold tumors and priming the antitumor immunity with less toxicity (4, 5).

Neutrophils, a type of polymorphonuclear (PMN) cells, have short lifespan and play an indispensable role in immune defense by their tumoricidal activity, granules release and etc. that are necessary for tumor suppression (6). Neutrophils regulate the microenvironment through pro- and anti-inflammatory cytokines expression; in turn, cytokines in the microenvironment mediate the function of neutrophils (7). Various subtypes of tumor-associated neutrophils (TANs) either promote or inhibit tumorigenesis, metastasis, and recurrence (8, 9).

TANs are distinguished from the potent immune-suppressive polymorphonuclear myeloid-derived suppressor cells (PMN-MDSCs) not by cell surface markers but only by functional characteristics (9). A recent study by Condamine et al. found that the lipid metabolism-related protein lectin-type oxidized LDL receptor-1 can distinguish PMN-MDSCs from neutrophils, yielding a potential target in medical oncology (10). However, in some cancer types, neutrophils have disparate roles in either supporting or suppressing the tumor cells proliferation, depending on the cancer types (11).

The contribution of neutrophils after low or high radiotherapy doses to patients’ metastasis-free survival (MFS) and local tumor control is still a matter of controversy. Elevated numbers of peripheral neutrophils result in radioresistance by activating the mitogen-activated protein kinase (MAPK) pathway (12, 13). Neutrophil depletion through antibodies or pharmacological approaches before radiotherapy boost cancer immune response of cancer patients (12). However, compelling evidence also indicates that fractionated radiation doses initiate neutrophil recruitment and promote antitumor immunity (14). These antitumor effects of neutrophils are amplified by concurrent administration of granulocyte colony-stimulating factor (G-CSF) and radiotherapy through PI3K/Akt/Snail signaling pathway activation (15, 16). Therefore, the distinction between “bad” and “good” neutrophils after low- or high-dose irradiation may pave the way to enhance “good” neutrophils and inhibit the “bad” neutrophils as a potential target to increase radiation efficacy (17, 18).

In this review, we will emphasize several phenotypes of neutrophils after fractionated low- or high-dose radiation, respectively and provide the potential target to enhance the combination therapy.

## 2 The functional versatility of neutrophils in response to conventional high- or low-dose irradiation therapies

In the early stages of human lung tumors, TANs compose up to 25% of cells from isolated tumor samples, indicating neutrophils’ indispensable role in tumor development and further progression (19). At the same time, crosstalk between T cells and neutrophils in this period encourages cytotoxic signaling expression on the surface of neutrophils, which fosters T cell proliferation (9, 19). Neutrophils also demonstrate functional diversity in the late stage of BALB/C mice bearing 4T1 mammary carcinoma. High-density neutrophils (HDN) from tumor-bearing mice prompt antitumor activity while low-density neutrophils (LDN) induce immunosuppressive properties (20, 21).

Neutrophils are divided into three major subtypes: pre-neutrophils, immature and mature neutrophils from previous reports, and these subtypes have various functional capacities (22). Neutrophil to lymphocyte ratio (NLR), an indicator and prognostic factor of progression-free and overall survival, a higher ratio is proportional to a lower immune response and more frequent relapsing rate in metastatic Non-small-cell lung carcinoma (NSCLC) (23). NLR provides an extensive range of cancer types with prognostic biomarkers after high- and low-dose irradiation treatment (23–25). In cervical cancer autochthonous mouse models of soft tissue sarcoma, elevated neutrophil numbers suppress the immune response and promote resistance to radiotherapy after the tumor receives a total 20 Gy *in situ* radiation (12). The accumulation of monocytic (M-)MDSCs in the tumor sites of MC38 tumor-bearing mice after 20 Gy irradiation stimulation results in radioresistance through STING pathway activation (26).

The effects of repeated radiation doses on positively or negatively regulating the immune can be influenced by the neutrophils that are infiltrated into tumors. Despite the immunosuppressive properties of neutrophils, neutrophils also participate in the first line of innate and adaptive immune response in tumor models after radiotherapy (14, 15, 27). Fractionated radiation of 8Gy leads to a remarkable increase of phosphorylated histone H2AX (γH2AX) which considerably induces CXCL1, CXCL2, and CCL5 inflammatory chemokine secretion and improves radiosensitivity. Neutrophils recruited by hypofractionated radiation facilitate mesenchymal-epithelial transition (MET) *via* the ROS-mediated PI3K signaling pathway and impel tumor elimination. A combination of high- or low-dose radiotherapy and granulocyte colony-stimulating factor (G-CSF) enhances radiosensitivity and antitumor activity with anticancer functional neutrophils (14, 15, 28, 29).

## 3 Neutrophils are reprogrammed in the tumor microenvironment after either low- or high-dose radiotherapy

Neutrophils are a critical component of the innate immune system, the pro- or anti-inflammatory functions of neutrophils depends on cytokines stimulation in the TIME (30–32). MDSCs, for example, is a type of the immunosuppressive neutrophils that involve in tumor progression in numerous cancer types after pathogen activation (33). Antitumor neutrophils can be regulated by ICAM-1 and TNFα, leading to reduced neutrophil extracellular traps (NETs) and improved radiotherapy response, resulting in prolonged survival in cancer patients (8, 9, 34, 35). With a better understanding of the crosstalk between neutrophils and the TIME, patients could benefit from improved diagnosis, more effective immune protection strategies, and targeted therapies.

## 4 Antitumor effects of neutrophils after high-dose or low-dose irradiation

### 4.1 Type 1 tumor-associated neutrophils

Similar to tumor-associated macrophages (TAMs), neutrophils undergo a reprogramming process under the stimulation of chemokines, tumor necrosis factors, colony-stimulating factors (CSFs), and interferons (IFN), which results in non-identical polarized phenotypes. Some are antitumor (N1 TANs), while others promote tumor progression and metastasis and pro-tumor TANs (36). However, there is currently no definitive method for identifying N1 TANs and N2 TANs (pro-tumor TANs) based on specific functional characteristics (37).

IFN-α and IFN-β, also referred as type 1 IFN, are considered as potential anticancer agents to maintain immune system surveillance capabilities. IFN- β can polarize neutrophils into an antitumor N1 phenotype (38). The presence of IFN-β in the early stage of tumor development restricts angiogenesis and backing neutrophils to antitumor N1 phenotype. This conversion leads to an increase in cytotoxic T cells and suppression of tumor cell proliferation, leading to improved outcome of cancer patients (36, 38). Antitumor N1 TANs have been shown, through immunohistochemical analysis in various cancer types, to promote leukocyte recruitment by producing cytotoxic reactive oxygen species (ROS), tumor growth inhibitor matrix metalloproteinase (MMP)-8, Fas-ligand for antibody-dependent cell-mediated cytotoxicity (ADCC), and multiple cytokines. These mechanisms result in similar antitumor effects regardless of whether high or low doses of radiation are used (36, 39, 40).

Several syngeneic mouse tumor models were utilized to gain deeper insight into the early immunological effects of different doses of radiation on neutrophils in TIME. In EG7-bearing C57BL/6 mice, which is a radiosensitive syngeneic graft tumor model, showed remarkable impact on tumor viability with 1.3Gy radiation and enhanced the antitumor effects through CD11b^+^Gr-1 high^+^ neutrophils infiltration. However, 4T1-bearing Balb/c mice required a high dose (15 Gy) to reach similar anti-neoplastic influence through heightened production of ROS neutrophils (15).

### 4.2 Antitumor neutrophil extracellular traps

Neutrophils play vital roles in protecting the host against infections through NETs. They immobilize and eliminate invading pathogens by activating the downstream ROS pathway and inducing chromatin decondensation promoters such as protein-arginine deiminase type 4 (PAD4), myeloperoxidase (MPO), and neutrophil elastase (NE) (41, 42). Although the mechanisms by which NETs are involved in the TIME and their potential anti- or pro-neoplastic activation have been explored for decades, it remains unclear how NETs regulate different types of tumor progression and elicit radioresistance or radiosensitivity after different doses radiation. One proposed mechanism by which NETs may promote tumor growth is the induction of chronic inflammation in the TIME. Moreover, the DNA in the NETs can act as a danger signal to trigger the activation of innate immune cells and further exacerbating inflammation in the TIME. However, reports also mentioned the anti-tumor effects of NETs. For example, NETs can trap circulating tumor cells and prevent tumor dissemination, which can reduce the tumor metastasis (11, 39, 42).

Histology analysis of adenocarcinoma patients demonstrates that the concentration of NETs reaches a peak in tumor sites in response to tumor cells, and neutrophil infiltration decreases gradually from tumor tissues to distal sites. *In vitro* evidence confirms that cultured Caco-2 colorectal adenocarcinoma cells and acute myeloid leukemia (AML) undergo apoptosis processes when encountering NETs (43). Toll-like receptors (TLRs) stimulate the formation of NETs (44). Furthermore, specific TLRs selectively produce corresponding NETs structurally and functionally distinct, which offers a promising foundation for further application in disease intervention (45). Co-culture with endothelial cells promote NETs formation, causing damage to tumor-dependent blood vessels and slowing tumor growth; this antitumor effect could be abrogated *via* NADPH oxidase inhibition (46). Histones, a component in NETs, can trigger host cell cytotoxicity, suggesting that NETs could be a promising target to increase tumor-killing efficacy (47).

## 5 Pro-tumor effects of neutrophils after high-dose and low-dose irradiation

Despite the extensive literature on the antitumor functions of TANs mentioned above in modified tumor cell lines or after receiving specific therapies, there are a substantial number of studies suggest their pro-tumor roles (48, 49). Increasing reports support the contribution of neutrophils in tumor progression through tumor angiogenesis, chemokine and cytokine release in TIME, which induce the pro-cancer role of neutrophils and resistance to radiotherapy (12, 26, 50). Pro-tumor TANs are associated with tumor metastasis, tumor cell proliferation, and a high frequency proportion of relapse. Neutrophil depletion through antibodies or genetic methods increases radiotherapy sensitivity (12). Neutrophils support the extravasation of disseminated carcinoma cells and inhibit intraluminal cell clearance mediated through NK cells; furthermore, such pro-cancer neutrophils show an extended life span (51–53).

### 5.1 Myeloid-derived suppressor cells

A subtype of immature and mature neutrophils, identified as inhibitory immune cells partly through promoting the proliferation of regulatory T cells (Tregs) and macrophage differentiation at pre-metastatic niches, are known as MDSCs (31, 54). Prostaglandin E2 (PGE-2), an essential cell growth and regulatory factor whose receptor is expressed on the surface of MDSCs, encourages differentiation of Gr1^+^CD11b^+^ MDSCs. PGE-2 inhibits the capacity of Th1, CTL, and NK cell and enhances Th2, Treg, tumor-infiltrating T helper type 17 (Th17), and tumor-infiltrating helper T cells inhibitory properties. PGE-2-deficient BALB/c mice display lower MDSCs expression and delayed tumor growth compared with wild-type mice when incubated with 4T1 tumor cells (55, 56). Activation of CXCL12/CXCR4 pathway leads to PEG-2-dependent accumulation of MDSCs, which in turn migrating *via* COX2 and promote tumor progression in ovarian cancer (57). Previous findings demonstrated that specific COX2 inhibitors, nonsteroidal anti-inflammatory drugs and other agents could be further developed as potential anticancer production by reducing PGE-2 synthesis or increasing neutrophil superoxide anion production to delay tumor progression and reduce metastasis (58, 59).

Radiation induces the infiltration of MDSCs into different types of tumors, with either low- or high- dose radiation (54, 60). The development of bone marrow-derived myeloid cells, including TAM and MDSCs, is dependent on CSF1/CSF1 receptor (CSF1R) (33, 61, 62). In prostate cancer, both multiple fractions of 3 Gy or a single 30Gy *in situ* radiation induce a systematic increase of MDSCs and CSF1/CSF1R in various organs through tumor-infiltrating myeloid cells (TIM) employment. Selective blockade of CSF1R suppresses MDSCs infiltration and facilitates MDSCs loss results in improved therapeutic outcomes (50, 63). In a report on MC 38 and CT26 tumor bearing mice, it reports that hypofractionated irradiation (>= 20Gy) suppresses the accumulation and infiltration of MDSCs. However, lower doses of irradiation tended to facilitate the recruitment of MDSCs into tumor (64, 65).

The influence of radiation on MDSCs infiltration and therapeutic outcomes are different between types of cancers. For example, a single dose of 25Gy radiation can recruit CD11b^+^Gr-1^+^ MDSCs to infiltrate tumors and affects last longer than 14 days in TRAMP-C1 intramuscular tumor model (66). However, there are significant differences between tumors and radiation doses. In high grade gliomas (HGG), a single dose of 4Gy radiation downregulates M2 TAMs and M-MDSCs and encourages T cell proliferation (67). Similar findings were shown in intracranial CT2A subcutaneous mouse model, where nanoparticles-based fractionated 2Gy repolarizes M2 pro-tumor phenotype to M1 antitumor phenotype and boosts ROS generation (66). High-dose radiation, which is over 45 Gy, can have various effects on MDSCs population in head and neck cancer patients (68).

Previous reports also suggest that MDSCs are responsible for abscopal effects inhibition. A melanoma patient who received radiotherapy showed decreased MDSCs concomitant with abscopal effects, suggesting a manageable way to investigate the relationship between radiation-induced MDSCs and tumor abscopal effects (69).

**Figure 1:**
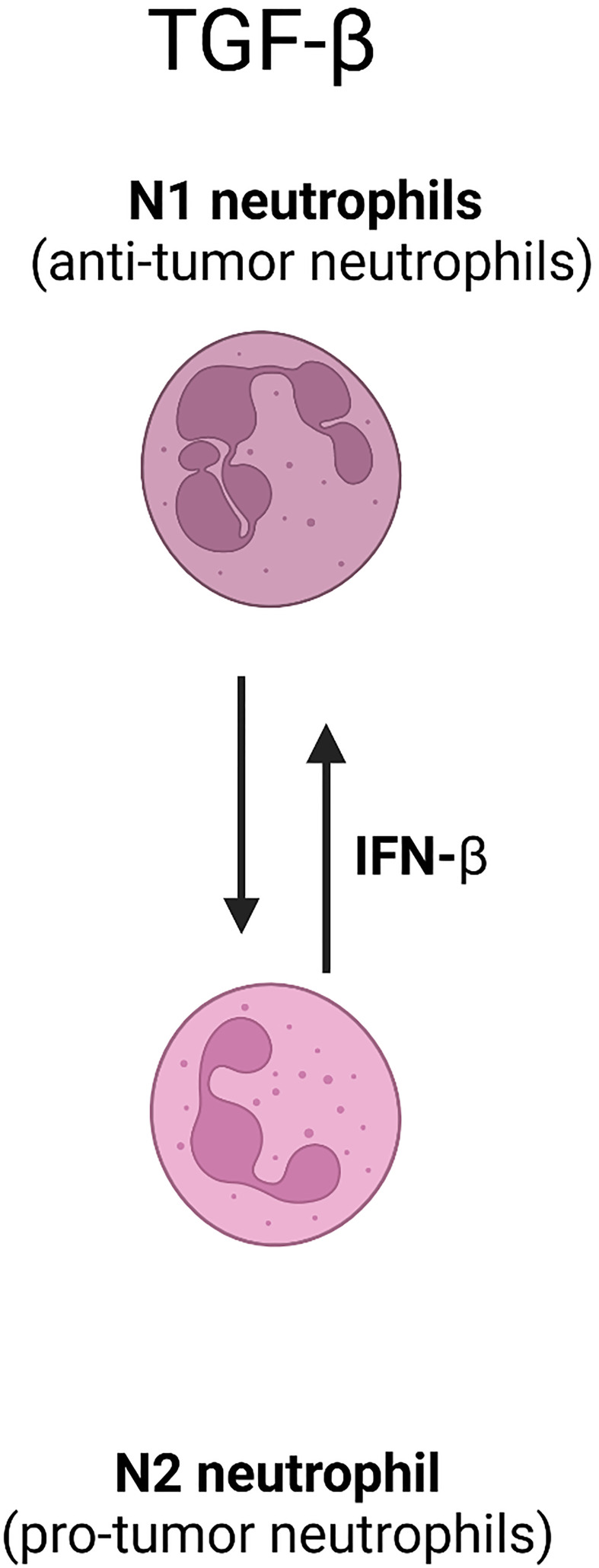
The involvement of TGF-β in neutrophil polarization.

The depletion of MDSCs, specifically targeting the crosstalk between MDSCs and NK cells, has been shown to mediate immunosuppressive influence and inhibit tumor growth after no more than 0.2Gy LDRT (70). Moreover, TGF-β secretion by MDSCs and N2-type TANs activation damage NK cells after receiving low radiation (63, 70) (**Figure 1**). The literature mentioned above reveals that the MDSCs which receives lose-dose and high-dose irradiation therapy establish various, and this yields a potential target to increase MDSC-based antitumor immunity in combination with different doses radiation with specific timing.

### 5.2 NETs formation

Neutrophils can prevent fungal and bacterial cells from invading through degranulation and nuclear chromatin expulsion and forming the NETs (42). However, in the context of radiation therapy, formation of NETs after radiation therapy can facilitate tumor progression, and its inhibition post-radiation improves the therapeutic outcome and overcomes radioresistance in syngeneic bladder cancer mode in a TLR-4 dependent manner (71). Tissue damage caused by radiation before tumor formation promotes Notch pathway activation, which leads to neutrophils’ recruitment to tumor sites and subsequent cancer metastasis (72).

Extracellular DNA accumulation is a marker of NET formation, and DNA components of NETs (DNA-NETs) promote cancer metastasis. DNase, which destroys DNA scaffolds through CCDC225, has been shown to abrogate NET-mediated metastasis (73, 74). Preclinical models have shown that NETs inhibition through PAD4 inhibitors (citrullination) increases sensitivity to anti-PD-1 and anti-CTLA 4 and achieve significant therapeutic efficacy (73).

Mitochondrial biogenesis and tumor cell proliferation have been found to be correlated with NETs formation and PAD4 expression in human cell lines (75–77). High dose of radiation (10 Gy) induces NETs formation in tumors, which capture tumor cells and shield them from detection by CTLs to facilitate tumor cell metastasis to distal sites (50). PAD4-KO tumors have the properties of elevated apoptosis, mitochondrial membrane potential and less ATP production, indicating the potential target for clinical appilicability (50, 75). However, a study also shows low-dose radiation (2Gy) has no influence on NETs formation in bladder cancer model (50). The involvement of NETs in radiation resistance demands further exploration.

### 5.3 N2 TANs

Substantial evidence suggests that functional disparity by factors in the TIME is the basis of the heterogeneity of neutrophils. For example, tumor releases IL-8, IL-10, PGE-2, and TGF-β to interstitially induce tumor progression N2 TANs and promote tumor growth and metastasis (12, 78). In addition, VEGF and TNF production from N2 TANs promote tumor vascularization and Matrix Metalloproteinases (MMP)-9 secretion, which participates in the tumor extracellular matrix reconstruction and contributes to subsequent tumor metastasis (9, 79, 80).

The tumor microenvironment and the patient’s overall TIME promote the conversion of neutrophils to the N2 TANs phenotype, as previously described that the absence of IFN-β stimulation leads to N2 TANs to promote tumor growth (36). Moreover, neovascularization was accompanied by increased invading N2 TANs that expressed more VEGF and CXCR4 (19, 81).

**Figure 2:**
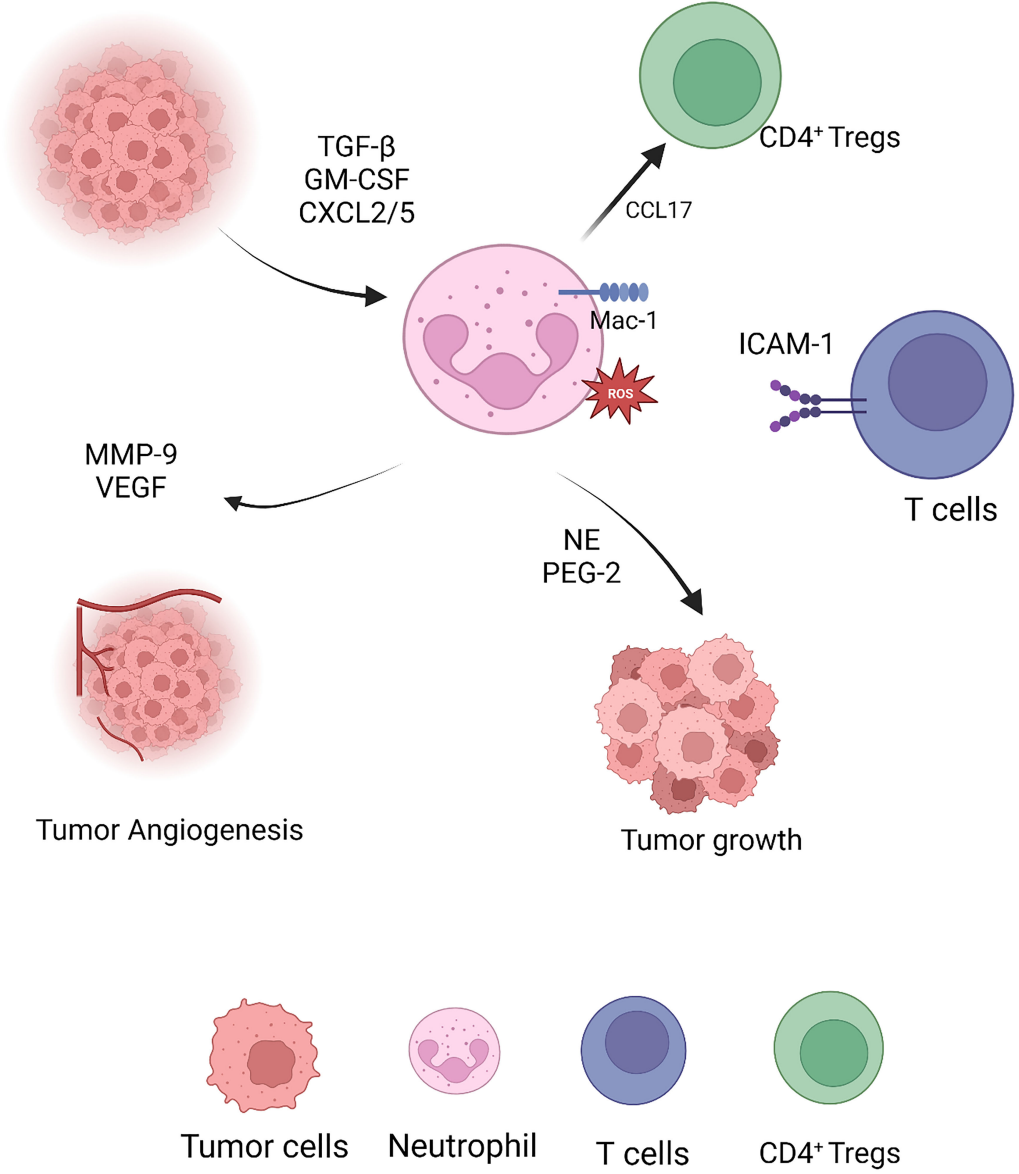
Pro-tumor capacities of neutrophils.

In contrast, differentiated N2 TAN secretes copious hydrolases, cytokines and chemokines to reinforce the immunosuppressive state of the TIME and promote tumor proliferation and migration. Tumor cells, pro-neoplastic neutrophils and even tumor-derived fibroblasts secrete MMP-9 to mediate the degradation of the tissue basement membrane type IV collagen to promote tumor growth and stimulate VEGF to promote vascularization (82, 83) (**Figure 2**).

N2-type neutrophils in tumor tissues stimulate Th17 proliferation and differentiation through CCL-20, IL-23 secretion and inhibition of TNF-α production, which in turn strengthens the immunosuppressive effects of Th17 and inhibit antitumor effects of CD4^+^ Th1 to negatively regulate functional homeostasis to tumor onset (14, 84–86). In the preclinical transgenic lung tumor mouse model, abnormal neutrophil accumulation correlated with an N2-like SiglecF^pos^ and Ly-6G downregulation. Furthermore, incomplete neutrophil deletion mediated with Ly-6G along with the radiotherapy retards tumor growth and triggers durable tumor regression (82).

## 6 Conclusion and perspectives

Radiation therapy induces a systematic immune response in cancer patients and improves survival. However, the cytotoxic effects are not persistent and patients develop radioresistance after several months. Therefore, more concurrent administration strategies are needed to refine radiotherapy.

Factors determine the function and polarization of neutrophils in the TIME; thus how the signalings and chemokines are involved in inducing the chemotaxis has been explored for several decades but still needs more elucidation. Neutrophil-targeted therapies offer a promising therapeutic route to strengthen therapeutic effects in a wide range of cancers from preclinical to clinical cancer patients. In multiple cancer patients’ histology samples, NLR is regarded as a reliable and accessible biomarker for predicting prognosis as cancer progresses. Low- or high radiotherapy slows tumor growth, interrupts the tumor cell cycle and releases neo-antigen from tumor cells to recruit immune cells and reinforce anti-neoplastic neutrophils and or dampen pro-tumor neutrophils to accelerate tumor shrinkage or undermine radioresistance, the mechanisms are still unclear. Chemotherapies or ICI and different doses of synergistic radiotherapy strategies render cancer patients responsive to radiation therapy to amplify immune responses and extend the cytotoxic effects of functional T cells. Cancer patients are benefited from combinational therapy with tumor shrinkage and extended survival. However, owing to the TIME and tumor gene mutation burden (TMB) in multiple cancer types, further research is necessary to better illustrate the interactions between neutrophils and high- and low-dose radiation therapy.

In addition, limited documents elucidate the appropriate application time and doses of radiation when combined with neutrophil-target therapies across various cancer classifications, which highlights the necessity for expanding our understanding of the precise underlying mechanisms of how tumor-induced cytokines and chemokines modify neutrophils to inhibit immunosuppressive neutrophils and extend immunotoxic neutrophils.

## Author contributions

Conceptualization, JH and MW. Writing—review and editing JH, MW, MP, YW and YZ. All authors contributed to the article and approved the submitted version.
